# Spatial Variation of Child Stunting and Maternal Malnutrition after Controlling for Known Risk Factors in a Drought-Prone Rural Community in Southern Ethiopia

**DOI:** 10.5334/aogh.3286

**Published:** 2021-08-18

**Authors:** Mehretu Belayneh, Eskindir Loha, Bernt Lindtjørn

**Affiliations:** 1 School of Public Health, College of Medicine and Health Sciences, Hawassa University, Hawassa, Ethiopia; 2 Centre for International Health, University of Bergen, Bergen, Norway; 3 Chr. Michelsen Institute, Bergen, Norway

## Abstract

**Background:**

Globally, understanding spatial analysis of malnutrition is increasingly recognized. However, our knowledge on spatial clustering of malnutrition after controlling for known risk factors of malnutrition such as wealth status, food insecurity, altitude and maternal characteristics is limited from Ethiopia. Previous studies from southern Ethiopia have shown seasonal patterns of malnutrition, yet they did not evaluate spatial clustering of malnutrition.

**Objective:**

The aim of this study was to assess whether child stunting and maternal malnutrition were spatially clustered in drought-prone areas after controlling for previously known risk factors of malnutrition.

**Methods:**

We used a community-based cohort study design for a one-year study period. We used SaTScan software to identify high rates of child stunting and maternal malnutrition clustering. The outcome based was the presence or absence of stunting and maternal malnutrition ([BMI] <18.5 kg/m^2^). We controlled for previously known predictors of child stunting and maternal malnutrition to evaluate the presence of clustering. We did a logistic regression model with declaring data to be time-series using Stata version 15 for further evaluation of the predictors of spatial clustering.

**Results:**

The crude analysis of SaTScan showed that there were areas (clusters) with a higher risk of stunting and maternal malnutrition than in the underlying at risk populations. Stunted children within an identified spatial cluster were more likely to be from poor households, had younger and illiterate mothers, and often the mothers were farmers and housewives. Children identified within the most likely clusters were 1.6 times more at risk of stunting in the unadjusted analysis. Similarly, mothers within the clusters were 2.4 times more at risk of malnutrition in the unadjusted analysis. However, after adjusting for known risk factors such as wealth status, household food insecurity, altitude, maternal age, maternal education, and maternal occupation with SaTScan analysis, we show that child stunting and maternal malnutrition were not spatially clustered.

**Conclusion:**

The observed spatial clustering of child stunting and maternal malnutrition before controlling for known risk factors for child stunting and maternal malnutrition could be due to non-random distribution of risk factors such as poverty and maternal characteristics. Moreover, our results indicated the need for geographically targeted nutritional interventions in a drought-prone area.

## Introduction

Child and maternal malnutrition remain a persistent problem, resulting in substantial increases in overall morbidity and mortality in developing countries [1]. Globally, maternal and child malnutrition related factors contribute to approximately 35% of child deaths and 11% of the total global disease burden [1]. Thus, the global efforts to avert malnutrition continued through the Millennium Development Goals (MDGs) era, and the Sustainable Development Goals (SDGs) set targets with the aim of eliminating malnutrition by 2030 [2]. Similarly, policy and national nutrition programmatic efforts have been made in Ethiopia to tackle the continuing challenge of malnutrition [3]. Despite concerted efforts, the prevalence of malnutrition remains high in low and middle income countries, and the progress to reduce malnutrition remains slow [4]. The issue of malnutrition is a complex phenomenon and it is the result of a dynamic interaction between factors such as illness and insufficient intake of dietary energy, underlying causes such as food insecurity and lack of health services access, and basic causes such as household economy, education, occupation, political and environmental reasons [5,6].

To reduce health disparities, an understanding of the geographical distribution of health problems is increasingly recognized by the global health community. Accordingly, researchers are more often using spatial analysis of malnutrition as it is helpful to understand the complex nature of the geographical distribution of malnutrition [7,8]. Knowledge about spatial distribution of malnutrition can provide information about how to allocate resources to vulnerable areas, and to plan effective interventions [8].

Earlier studies on spatial analysis of malnutrition conducted in Ethiopia showed spatial clustering [9,10]. However, they did not evaluate the occurrence of spatial variation of malnutrition after controlling for known risk factors. To fill this knowledge gap, we designed this study to evaluate whether child stunting and maternal undernutrition were clustered or not, after controlling for known risk factors for malnutrition in the area. This study was a part of a large cohort study where we found that the risk of child malnutrition was associated with household characteristics such as poverty, education, occupation, household food insecurity and the risk of maternal malnutrition was higher among poor households and among older mothers. The households with malnourished mothers often had malnourished children. Moreover, the findings link both maternal malnutrition and child malnutrition with seasonal variation of household food insecurity among drought-prone rural farming communities [11,12].

The aim of this study was to assess whether child stunting and maternal malnutrition were spatially clustered in drought prone areas after controlling for known risk factors of malnutrition among children and their mothers.

## Methods and materials

### Study area

This study was conducted in Boricha, which is geographically located at 6º 46’N and 38º 04’E to 7º 01’N and 38º 24’E in southern Ethiopia. Boricha district is a drought prone area, approximately located 34 km south of Hawassa, the capital city of Southern Nations Nationalities of Peoples Region (SNNPR).

Sidama is the largest ethnic group in the district and more than 90% of the population lives in rural areas. Protestant Christianity is the main religion in the district and most of people make their living directly from subsistence farming and livestock rearing. Based on the 2007 national census, approximately 315,000 people lived in the district in 2017. The district has 39 rural kebeles (smallest administrative units), each with an average population of 1,000 to 6,000 people. In 2017, there was one government hospital, five public health centres, 39 health posts in the district. Each kebele has at least one health post staffed by two health extension workers who report to the health centre.

As Boricha is the drought prone area, malnutrition is the major health problem. Highest peak of acute malnutrition cases occurs between March and June during Belg rainy season, and the lowest occurrence of acute malnutrition occurs between September and December, following rains in March and June [11]. Moreover, the absence of rivers and far to reach to underground water serve as potential malnutrition sites. Thus, study area is known for drought prone and included in Safety Net Programme.

### Study design and period

This study is a part of a community-based cohort study conducted to evaluate child and maternal malnutrition at the community level. During 2017, all households were visited four times. The aim of our study was to measure the patterns and determinants of child stunting and child wasting, determine the risk factors of maternal malnutrition, and evaluate spatial clustering of child and maternal malnutrition.

#### Outcome and exposure variables

The main outcomes of the study were the spatial clustering of child stunting and maternal malnutrition. The main exposure variables were wealth, altitude, household food insecurity access, maternal education, maternal occupation, and maternal age. The aforementioned covariates were related to child and maternal malnutrition according to their significance level in the previous studies conducted in Boricha district and they are assumed to be non-randomly distributed geographically [11,12]. Thus, we want to find clusters that cannot be explained by these covariates.

### Study population and sampling

We employed two-stage sampling technique to select mother-child pairs. First, nine rural kebeles (smallest administrative unit) were randomly selected from 39 rural kebeles of Boricha district. Secondly, out of the nine rural kebeles, we recruited study subjects from gouts (villages) through cluster sampling technique.

This cohort had included 935 children between 6 months to 47 months and 892 biological mothers between 15–49 years old from the total of 897 households at the beginning of the study. Children from 6 months to 47 months were recruited to accommodate age increment due to a one-year follow-up period of time. See earlier publication for detailed description of the study population [11].

### Data collection

We utilized eighteen data collectors and three supervisors for data collection, who were familiar with the local context and fluent speakers of the local language (*Sidamu Afoo*). They were trained and conducted pre-test outside of the selected kebeles that had similar socio-economic characteristics. We collected on socio-demographic data such as maternal age, educational status of mothers and occupational status of mothers.

Each household had a unique code and geo-referenced using a handheld global positioning system (GPS) device (Garmin’s GPSMAP60CSx, Garmin International Inc., Olathe, Kansas, USA).

### Food insecurity and dietary diversity

Household food insecurity was assessed by using the Household Food Insecurity Access Scale (HFIAS) tool developed by the FANTA project and validated in different seasons of Ethiopia [13,14,15]. Food consumption was assessed by Household Dietary Diversity Score (HDDS) of 24 hours recall measurements. Twelve food groups were measured: Meat or Poultry, Eggs, Fish, Cereals, Root or tubers, Vegetables, Fruits, Pulses or legumes or nuts, milk and milk products, oil or fats, sugar or honey, and miscellaneous [13]. See earlier publication for detailed information [11].

### Wealth index

We constructed wealth index using principal component analysis [6]. Household assets-related variables such as type and number of herds, ownership of improved sanitation, type of fuel used for cooking food, materials used for construction of house wall, floor and roof, number of sleeping rooms, ownership of chair and mobile telephone. The principal component analysis Kaiser-Meyer-Olkin measure of sampling adequacy was 67% and significance level of below 0.001. The households were then ranked into five categories such as poorest, poor, medium, rich and richest.

### Child and maternal malnutrition

The term malnutrition denotes both to undernutrition and over-nutrition. It is a condition that results from eating a diet in which various nutrients are either an insufficient or excessive. However, this paper focuses on the maternal and child undernutrition aspect such as child stunting and maternal undernutrition based on BMI measurements.

Child anthropometric measurements were evaluated based on children’s height and recorded using Emergency Nutrition Assessment for Standardized Monitoring and Assessment of Relief and Transitions software (NutriSurvey for SMART, version 2011). Children up to 24 months of age were measured in a recumbent position using a length board to the nearest 0.1cm. Children who are able to stand unassisted measured in the standing position to the nearest 0.1cm. We used mother’s recall and memorable events to assess child age. Child stunting was defined as height-for-age Z-score of less than two standard deviation of the World Health Organization Child Growth Standards Median [16].

The presence of maternal malnutrition was defined as a body mass index [BMI] <18.5 kg/m^2^ [17]. The weight was recorded to the nearest 100 g using a digital SECA scale (SECA GmbH, Germany) and the height was measured with a locally prepared apparatus that had a 0.1 cm resolution.

### Data analysis

Data were double-entered and checked using EpiData v. 3.1 (Odense, Denmark), and transferred to STATA 15 (StataCorp, College Station, TX) for further cleaning and analysis. Descriptive statistics such as frequency counts, percentages, means and 95% CI were used to summarize the data. For the anthropometric measurements we deleted extreme values of child stunting (height-for-age Z-scores greater than six or less than minus six) which represented 4.9% of the measurements. Similarly, we deleted cases with improbable heights (about 1%).

Data visualization was done using ESRI ArcMap 10.4.1 (ESRI, Redlands, CA, USA) software. The coordinates’ projection was defined using Universal Transverse Mercator Zone 37°N and World Geodetic system 1984. We used SaTScan version 9.7 software (Free software, Kulldorff’s spatial scan statistics) and specified three Microsoft Excel files (case, population, and coordinates) as an input data to identify locations of statistically significant clusters after controlling for risk factors. Kulldorff’s spatial scan statistics was used to identify statistically significant clusters (purely spatial) of high stunting and maternal malnutrition ([BMI] <18.5 kg/m^2^) rates using a discrete scan statistics of Poisson probability model [18]. Scan statistics computed data across space through a circle for space and covered the entire period [18].

Spatial analysis used a circular window shape that was performed with maximum spatial cluster size as a 50% of the population at risk; 50% was the upper limit for SaTScan clusters and did not account SaTScan clusters of the population greater than 50%. The circular window with the maximum log likelihood was considered as the most likely cluster area if P-value < 0.05 and the remaining clusters were reported as secondary cluster if they are geographically non-overlapping windows with the P-value < 0.05 [18]. The maximum number of Monte Carlo replications was 999 and the minimum number of two cases was required for high rates of clusters. SaTScan Version 9.7 calculated the P-values by using the combination of Monte Carlo, sequential Monte Carlo, and Gumbel approximation [18].

To evaluate whether there is clustering or not when the known risk factors for clustering in the study area are controlled, we compared crude analysis of SaTScan with the adjusted analysis after including covariates in the SaTScan Version 9.7. The dependent variable was a binary outcome (yes/no) based on the presence or absence of stunting and maternal malnutrition [BMI] <18.5 kg/m^2^. We considered the known predictor variables such as wealth index, food insecurity, altitude, maternal education, maternal occupation and maternal age. These covariates were selected based on their significant association with child stunting and maternal malnutrition ([BMI] <18.5 kg/m^2^) according to previous research conducted in Boricha district [11,12].

Although identifying the presence of clustering after controlling for known risk factors is the primary objective, we also identified the effects of the risk factors for the observed clustering [19]. Hence, we did a logistic regression model with declaring data to be time-series using Stata version 15 for further evaluation of the predictors of spatial clustering. The time setting of the model used season of the year as time variable, and child code was considered as panel ID variable. The model construction used an exchangeable correlation matrix, and a main effect term builder. The stunted children identified within the spatial cluster were compared with stunted children outside the cluster. The presence or absence of differences of risk factors between the groups (stunted children in the cluster versus stunted children outside the cluster) could give information about the underlying risk factors that may be responsible for the observed clustering. The potential risk factors considered were wealth status, altitude, household food insecurity, maternal age, maternal education, and maternal occupation. However, because of the very wide cluster radius size in the spatial cluster of maternal malnutrition, we limited our analysis only for stunting [20].

### Ethical consideration

We had secured ethical approval from Institutional Review Board (IRB) at Hawassa University (Ref. No: IRB/001/09, Date: 13/09/2016) and the Regional Committee for Medical and Health Research Ethics, Western Norway (ref: 2016/1631/REK Vest) before we had started data collection. All study participants were informed about the benefit and harm of the study, and then informed written consent was obtained from all study participants. Those who could not sign on used their thumb print.

### Acknowledgements

The authors thank the study subjects for their willingness to participate, and the data collectors, supervisors, and Boricha district authorities for their positive input.

## Results

### Characteristics of the study population

A total of 935 children residing in 897 households were visited for the four-consecutive time for the study. About 505 (54%) were boys and 427 (46%) were females. Most of our respondents (99%; 892 of 897) were biological mothers of children. The mean age of respondents was 28 years. About 50% (445 of 887) of mothers were illiterate. Occupationally, 770 of 889 (87%) of mothers were housewives and farmers. The average family size of the study households was five persons. See ***Table 1*** describes the characteristics of the study population by the nutritional status.

**Table 1:** The nutritional status by the selected baseline study characteristics, among children and their mothers in the Boricha district of Southern Ethiopia; March 2017.


BASELINE CHARACTERISTICS	CHILD MALNUTRITION (N = 935 CHILDREN: 3312 MEASUREMENTS)		MATERNAL MALNUTRITION (N = 892 MOTHERS: 3179 MEASUREMENTS)	

		STUNTING		THINNESS

	MEAN (95% CI)	% WITH Z-SCORE < –2	MEAN (95%CI)	BMI <18.5 KG/M^2^

Child age (in months)				

6–11	–0.9 (–1.21, –0.64)	35 (25.2%)	19.9 (19.48, 20.34)	40 (30.8%)

12–23	–1.5 (–1.63, –1.34)	316 (43.5%)	19.6 (19.47, 19.80)	214 (31.2%)

24–35	–1.6 (–1.72, –1.47)	328 (39.7%)	19.85 (19.67, 20.03)	226 (28.3%)

36 and above	–1.8 (–1.93, –1.76)	729 (44.9%)	20.3 (20.19, 20.44)	351 (23.5%)

Child sex				

Male	–1.7 (–1.79, –1.62)	791 (44.1%)	20.1 (19.97, 20.20)	443 (25.5%)

Female	–1.6 (–1.71, –1.53)	617 (40.6%)	20.0 (19.84, 20.10)	410 (28.5%)

Mother’s age (in years)				

19 and below	–1.3 (–1.98, –0.65)	2 (33.3%)	24.7 (23.17, 26.23)	0

20–29	–1.6 (–1.65, –1.49)	742 (39.3%)	20.4 (20.30, 20.52)	374 (20.8%)

30–39	–1.8 (–1.86, –1.66)	607 (46.3%)	19.5 (19.35, 19.62)	447 (35.4%)

40 and above	–1.9 (–2.19, –1.52)	34 (42.0%)	19.8 (19.16, 20.42)	28 (33.3%)

Household food insecurity				

Food secured	–1.7 (–1.83, –1.52)	229 (40.2%)	20.5 (20.25, 20.69)	124 (21.7%)

Food in-secure	–1.7 (–1.74, –1.60)	1,174 (43.1%)	19.9 (19.84, 20.03)	729 (28.1%)

Household dietary diversity				

<=3	–1.5 (–1.64, –1.29)	189 (39.5%)	20.2 (19.98, 20.42)	116 (24.5%)

>4	–1.8 (–1.84, –1.69)	878 (44.6%)	20.0 (19.86, 20.10)	536 (27.4%)

Wealth quantiles				

Poorest	–1.9 (–2.03, –1.72)	331 (50.2%)	19.7 (19.48, 19.85)	216 (31.5%)

Poor	–1.6 (–1.78, –1.51)	293 (42.5%)	20.0 (19.79, 20.17)	186 (28.8%)

Medium	–1.7 (–1.79, –1.51)	258 (40.8%)	20.3 (20.14, 20.53)	120 (20.6%)

Rich	–1.7 (–1.81, –1.55)	290 (42.8%)	20.0 (19.77, 20.18)	180 (29.3%)

Richest	–1.4 (–1.57, –1.32)	224 (35.2%)	20.2 (20.03, 20.41)	155 (24.6%)

Family size				

<=5	–1.7 (–1.73, –1.57)	804 (41.9%)	20.1 (20.01, 20.23)	447 (24.5%)

>5	–1.7 (–1.81, –1.61)	532 (44.1%)	19.9 (19.78, 20.08)	355 (30.6%)

Mother’s education				

Primary	–1.6 (–1.67, –1.49)	581 (39.1%)	20.0 (19.84, 20.11)	417 (29.1%)

Secondary	–1.0 (–1.27, –0.78)	39 (26.0%)	20.7 (20.35, 21.14)	20 (14.4%)

Non formal education	–1.8 (–1.88, –1.70)	780 (47.1%)	20.0 (19.91, 20.14)	415 (26.2%)

Occupational status of mother				

Housewife & farmer	–1.7 (–1.77, –1.63)	1233 (43.5%)	20.0 (19.88, 20.07)	764 (27.8%)

Merchant/employed	–1.4 (–1.56, –1.25)	136 (35.1%)	20.4 (20.18, 20.70)	76 (22.1%)

Other	–1.9 (–2.32, –1.48)	38 (47.0)	20.4 (19.92, 20.82)	12 (15.4%)

Father’s educational status				

Primary	–1.7 (–1.75, –1.59)	692 (41.5%)	20.0 (19.83, 20.07)	456 (28.4%)

Secondary and above	–1.3 (–1.45, –1.12)	120 (30.2%)	20.3 (20.00, 20.53)	80 (22.3%)

No formal education	–1.8 (–1.87, –1.65)	581 (47.4%)	20.1 (19.93, 20.22)	313 (26.2%)

Father’s occupational status				

Farmer	–1.8 (–1.85, –1.69)	923 (44.8%)	20.0 (19.92, 20.15)	538 (27.6%)

Merchant	–1.4 (–1.51, –1.30)	318 (35.6%)	19.8 (19.68, 20.02)	257 (29.4%)

Others	–1.8 (–1.96, –1.58)	159 (46.4%)	20.5 (20.26, 20.75)	54 (16.3%)

Residency				

Sadamo Dikicha	–1.6 (–1.81, –1.45)	142 (40.1%)	18.6 (18.35, 18.93)	199 (52.0%)

Alawo Siso	–1.0 (–1.16, –0.84)	93 (25.9%)	20.1 (19.89, 20.39)	78 (25.9%)

Fulasa Aldaada	–2.3 (–2.62, –2.06)	177 (61.5%)	22.0 (21.73, 22.24)	27 (7.6%)

Aldaada Deela	–1.6 (–1.81, –1.46)	137 (37.9%)	20.0 (19.76, 20.25)	89 (25.7%)

Kitawo Dambie	–2.2 (–2.38 –2.06)	198 (54.3%)	18.8 (18.56, 19.01)	170 (50.0%)

Gonowa Bulano	–2.2 (–2.37, –2.06)	233 (59.3%)	20.1 (19.85, 20.33)	66 (22.3%)

Sadamo Challa	–1.4 (–1.57, –1.32)	122 (30.1%)	19.9 (19.69, 20.08)	80 (20.8%)

Qonsore haranja	–0.9 (–1.11, –0.78)	108 (26.1%)	21.3 (21.05, 21.49)	35 (9.0%)

Hanja Gooro	–1.8 (–1.97, –1.55)	198 (52.9%)	19.5 (19.31, 19.68)	114 (29.6%)


### Nutritional status of child and their mothers

We found that the prevalence of stunting and maternal malnutrition varied among the nine kebeles. The highest prevalence of stunting (62%) and was recorded in Fulasa-Aldaada kebele. The highest prevalence of maternal malnutrition (52%) recorded in Sadamo-Dikicha kebele. The prevalence of stunting increased as the age of the child increased. See ***Table 1*** describes the characteristics of the study population by the nutritional status.

### Spatial clustering of stunting

The crude analysis of SaTScan showed that, there were areas with higher risk of stunting than in the underlying at-risk populations. The most likely and secondary significant spatial clusters for stunting were identified in the north and north-eastern part of the study area.

Children identified within the most likely clusters were 1.6 times more at risk of stunting than in the underlying at-risk populations in the unadjusted analysis. However, covariate adjusted SaTScan analysis showed that clustering disappeared. See ***Table 2*** for more information.

**Table 2:** Purely spatial scan statistics of the significant clusters for stunting among children under the age of five years, Boricha, Southern Ethiopia, 2017.


	MOST LIKELY CLUSTERS	

	UNADJUSTED SATSCAN ANALYSIS	ADJUSTED SATSCAN ANALYSIS

Number of locations	166	–

Coordinates	6.989467 N, 38.286050 E	7.001650 N, 38.279983 E

Radius (km)	4.71 km	0 km

Population (No. of children)	614	4

Number of cases	361	3

Expected cases	258.67	2.00

Annual cases per 100,000	70 177.6	75 426.1

Observed/expected cases	1.40	1.50

Relative risk	1.55	1.50

Log likelihood ratio	23.36	0.216786

P-value	P < 0.001	P = 1.00

	**SECONDARY CLUSTERS**	

	**UNADJUSTED**	**ADJUSTED**

Number of locations	99	–

Coordinates	6.938250 N, 38.358983 E	–

Radius (km)	2.96 km	–

Population (No. of children)	318	–

Number of cases	201	–

Expected cases	133.97	–

Annual cases per 100,000	75 444.7	–

Observed/expected cases	1.51	–

Relative risk	1.60	–

Log likelihood ratio	16.531033	–

P-value	P < 0.001	–


### Spatial clustering of maternal malnutrition based on maternal BMI

The crude analysis of SaTScan showed that, there were areas with higher risk of maternal malnutrition than in the underlying at-risk populations. Mothers within the most likely clusters were 2.4 times more at risk of malnutrition in the crude analysis. However, covariate adjusted SaTScan analysis did not show spatial variation of maternal malnutrition. See ***Table 3*** for more information.

**Table 3:** Purely spatial scan statistics of the significant clusters for maternal BMI in the Boricha, Southern Ethiopia, 2017.


	MOST LIKELY CLUSTERS	

	UNADJUSTED SATSCAN ANALYSIS	ADJUSTED SATSCAN ANALYSIS

Number of locations	84	–

Coordinates	6.930917 N, 38.936267 E	6.923550 N, 38.288100 E

Radius (km)	62.16 km	0 km

Population (No. of children)	329	4

Number of cases	182	3

Expected cases	87.21	1.80

Annual cases per 100,000	66,029.1	52,731.9

Observed/expected cases	2.09	1.67

Relative risk	2.41	1.67

Log likelihood ratio	45.837737	0.333394

P-value	P < 0.001	P = 1.00

	**SECONDARY CLUSTER**	

	**UNADJUSTED**	**ADJUSTED**

Number of locations	56	–

Coordinates	6.913650 N, 38.295950 E	–

Radius (km)	1.57 km	–

Population (No. of children)	215	–

Number of cases	121	–

Expected cases	56.99	–

Annual cases per 100,000	67 174.9	–

Observed/expected cases	2.12	–

Relative risk	2.33	–

Log likelihood ratio	29.984131	–

P-value	P < 0.001	–


### Risk factors identification for spatial clustering

In this analysis we aimed to provide further insights into risk factors for the clustering of stunting and to evaluate the effects of risk factors for the observed crude spatial clustering. Accordingly, we found significant differences with regard to factors such as poverty, maternal age, maternal education, and maternal occupation between stunting cases found within a crude spatial cluster and cases outside the cluster (***Table 4***).

**Table 4:** Risk factor for clustering of stunting among children under the age of five years in nine kebele, Boricha, Southern Ethiopia, 2017.


VARIABLES		CASES WITHIN IDENTIFIED SPATIAL CLUSTER		UNADJUSTED OR		ADJUSTED OR	

		YES (%)	NO (%)	OR	95% CI	OR	95% CI

Food insecurity	Secured	95 (48.2)	102 (51.8%)	1	Ref	1	Ref

	Mild food insecurity	90 (75.0%)	30 (25.0%)	3.22	1.96–5.31	2.89	1.72–4.89

	Moderate food insecurity	101 (50.5%)	99 (49.5%)	1.10	0.74–1.62	0.97	0.63–1.49

	Severe food insecurity	274 (36.4%)	478 (63.6%)	0.62	0.45–0.84	0.42	0.30–0.60

Wealth index	Poorest	173 (53.6%)	150 (46.4%)	1	Ref	1	Ref

	Poor	88 (35.6%)	159 (64.4%)	0.48	0.34–0.67	0.37	0.25–0.54

	Medium	65 (27.9%)	168 (72.1%)	0.34	0.23–0.48	0.25	0.17–0.37

	Rich	138 (52.7%)	124 (47.3%)	0.96	0.69–1.34	0.74	0.51–1.06

	Richest	96 (47.1%)	108 (52.9%)	0.77	0.54–1.09	0.60	0.40–0.91

Mother’s age (in years)		560 (44.1%)	709 (55.9%)	0.99	0.97–1.01	0.94	0.92–0.96

Mother’s education	Illiterate	356 (48.9%)	372 (51.1%)	1	Ref	1	Re

	Primary	196 (38.7%)	311 (61.3%)	0.66	0.52–0.83	0.39	0.29–0.53

	Secondary and above	8 (23.5%)	26 (76.5%)	0.32	0.14–0.72	0.18	0.07–0.48

Mother’s occupation	Farmers and housewife	501 (45.3%)	605 (54.7%)	1	Ref	1	Ref

	Employed/merchant	49 (38.0%)	80 (62.0%)	0.74	0.51–1.08	0.59	0.39–0.90

	Others	10 (29.4%)	24 (70.6%)	0.50	0.24–1.06	0.50	0.18–1.42

Altitude		560 (44.1%)	709 (55.9%)	1.00	0.99–1.003	1.00	0.99–1.003


Stunted children within an identified spatial cluster were about four times more likely to be from poorest households than those outside a cluster. Stunted children within an identified spatial cluster were more likely to be from younger and illiterate mothers, and often the mothers were farmers and housewives. These findings suggest that these factors are non-randomly distributed, and varies across cases identified within the cluster and cases identified outside the cluster.

## Discussion

We found that child stunting and maternal malnutrition were not spatially clustered in drought prone areas after we controlled for known risk factors of malnutrition. The spatial clustering of child stunting and maternal malnutrition could be due to non-random distribution of risk factors of malnutrition such as poverty, maternal age, maternal education, and maternal occupation.

Our unadjusted scan statistics demonstrated that, the spatial distribution of child and maternal malnutrition was non-random process, as has also been demonstrated by other countries [21,22], including Ethiopia [9,10]. Some of these findings assumed that the basis for area-level spatial variation in malnutrition is due to individual or household level characteristics, which is similar to our findings [22,23]. Thus, the absence of clustering after controlling for known risk factors of child and maternal malnutrition could improve our understanding of possible factors of malnutrition clustering. Moreover, this could have implication for targeting nutritional interventions, and to consider type of nutrition related interventions such as educational, agricultural or health care services.

However, there could be more risk factors that might be associated with both child and maternal malnutrition, and that could influence the spatial heterogeneity of child and maternal malnutrition. For example, maternal depression and malnutrition could influence child malnutrition [24,25]. Micronutrient deficiencies such as zinc, iron, iodine and vitamins are prevalent among women and children due to higher physiological demands, and again these deficiencies could lead to higher risk of infections [26,27,28]. The occurrence of infectious diseases such as respiratory illnesses, malaria and diarrhoea can affect child and maternal malnutrition [29,30,31,32,33,34]. Furthermore, complementary feeding practices, multiple births, premature births, short birth length, maternal care, low birth weight are associated with increased risk of childhood malnutrition [35,36,37,38,39,40]. Contextual factors such as beliefs and norms could also be risk factors for malnutrition [41]. Moreover, our cohort study only lasted for one-year, and it is well-documented that the stunting process could start in prenatal period. Thus, it is advisable to conduct studies over longer periods to identify more risk factors of growth faltering during prenatal and early life [42].

### Strengths of the study

Among some of the strengths of the study, the risk factor like food insecurity was collected in four different seasons along with the child and maternal malnutrition measurements. We used non-aggregated (individual data points) which could increase the spatial resolution of cluster detection [20]. Moreover, our study identified some risk factors of spatial clustering of child and maternal malnutrition in the drought prone area.

We had less than 10% of missed measurements (height-for-age Z-score and body mass index) due to loss to follow-up and outliers, which indicated that the impact of loss to follow and outliers was low. Moreover, our study population is a representative sample of the population, and the non-response bias was low. We performed a cohort study design, and thus our study can establish temporal associations. Moreover, our study had consistency with the previous studies regarding to local clustering of child stunting [10].

### Limitations of the study

The non-random distribution of risk factors not evaluated in our study could influence the spatial clustering of child and maternal malnutrition. However, we included the most common risk factors of child and maternal malnutrition. We opted to use a circular window in the spatial scan statistics rather than other windows to identify the significant clusters; however, the true clusters may be elliptic or rectangular. Scan statistics using circular windows cannot detect these shapes, unless all possible angles are considered, which is difficult to compute [18].

Nutritional epidemiology has been criticized for its inaccurate survey measurements of food consumption and reliance on observational associations [43,44,45]. Thus, one method of enhancing the validity of nutritional studies is to conduct randomized controlled trials [44]. Moreover, it is recommended to apply new technologies such as biomarkers and digital technologies for nutritional epidemiology studies [45].

In this study, measurement errors could be originated from the anthropometric recordings, recall bias, and social desirability bias. However, to minimize such biases, we trained data collectors and supervisors, conducted pre-tests, evaluated technical error measurements, and calibrated instruments. Moreover, we used memorable events to aid the mother’s recall of their children’s age and we also used short recall periods for variables related to household food consumption. Missed information regarding government support such as the safety-net programme could influence the findings of our study.

### Conclusions and recommendations of the study

In conclusion, child stunting and maternal malnutrition were not spatially clustered after we controlled for known risk factors for child stunting and maternal malnutrition, and these risk factors are probably not randomly distributed. The results of this study would be helpful for geographically targeting nutritional interventions and to consider type of nutrition related interventions such as educational, agricultural or health care services in a drought prone area.
